# Bioconversion of food waste by *Chrysomya megacephala* (Diptera: Calliphoridae) larvae: Potential for sustainable waste management and antimicrobial applications

**DOI:** 10.1371/journal.pone.0320747

**Published:** 2025-04-15

**Authors:** Pluemkamon Phuwanatsarunya, Nophawan Bunchu, Worasak Kaewkong, Tongjit Thanchomnang, Ketsarin Thipphet, Sophit Khanthawong

**Affiliations:** 1 Department of Microbiology and Parasitology, Faculty of Medical Science, Naresuan University, Phitsanulok, Thailand; 2 Centre of Excellence in Medical Biotechnology, Faculty of Medical Science, Naresuan University, Phitsanulok, Thailand; 3 Department of Biochemistry, Faculty of Medical Science, Naresuan University, Phitsanulok, Thailand; 4 Faculty of Medicine, Mahasarakham University, Maha Sarakham, Thailand; Universiti Teknologi Malaysia - Main Campus Skudai: Universiti Teknologi Malaysia, MALAYSIA

## Abstract

Addressing global food waste requires innovative and sustainable solutions. This study investigates the potential of *Chrysomya megacephala* (Diptera: Calliphoridae) larvae to convert food waste into valuable byproducts, while evaluating the antimicrobial properties of larval extracts. Under controlled laboratory conditions, the larvae reduced the weight of food waste by 21.87%, demonstrating their effectiveness in waste degradation. The optimal food waste-to-sawdust ratio was identified as 10:1. The bioconversion process resulted in 111.60-fold increase in larval biomass when reared on food waste and a 153.20-fold increase on fresh pork liver, highlighting their efficiency in converting protein-rich substrates. Larval extracts demonstrated significant antimicrobial activity against *Bacillus subtilis* and *Pseudomonas aeruginosa*, with minimum inhibitory concentrations (MICs) of 100 µg/ml. Proteomic analysis revealed proteins with potential antimicrobial and antioxidative properties. Furthermore, the extracts promoted cell growth *in vitro* without showing cytotoxic effects on HaCaT cell lines, suggesting potential applications in wound healing and infection control. These findings highlight the capacity of *C. megacephala* larvae to reduce food waste while generating antimicrobial agents, offering a sustainable approach to waste management with promising implications in medical applications.

## Introduction

The growing global population and rapid urbanization have led to an exponential increase in food waste, presenting significant environmental, economic, and public health challenges. The Food and Agriculture Organization (FAO) estimates that approximately 1.3 billion tons of food are wasted annually, accounting for nearly one-third of global food production [1]. Conventional food waste disposal methods, such as landfilling and incineration, contribute to greenhouse gas emissions, environmental degradation, and public health risks, including exposure to particulate matter (PM2.5) [2,3]. Furthermore, improper food waste management exacerbates issues such as water pollution, pathogen proliferation, and the inefficient use of valuable organic resources [4]. These challenges underscore the urgent need for sustainable and innovative waste management solutions.

Simultaneously, the rising demand for animal feed, driven by increasing global meat consumption, emphasizes the need for alternative and sustainable protein sources. Traditional animal feed production is constrained by limited arable land, water scarcity, and the high environmental impact of crop cultivation [5]. Insects have emerged as a promising alternative, offering an efficient and resource-conscious method for waste bioconversion and the generation of high-quality protein for animal feed. Among these, blow fly larvae, including those of *Chrysomya megacephala* (Diptera: Calliphoridae), present an alternative with distinct advantages. Unlike *Musca domestica* (Diptera: Muscidae) and *Hermetia illucens* (Diptera: Stratiomyidae), which have been extensively studied for waste bioconversion, *C. megacephala* remains underexplored despite its natural adaptation to decomposing protein-rich organic waste [6,7].

In this context, *C. megacephala*, a blow fly species with a global distribution, presents a unique and underexplored opportunity for sustainable food waste management. Known for its adaptability to diverse environments, including urban and rural settings, *C. megacephala* naturally thrives on protein-rich organic waste [8], making it a suitable candidate for bioconversion applications. Beyond its role in organic matter decomposition, this species also exhibits biomedical potential, particularly in antimicrobial compound production. Previous research has confirmed the production of antimicrobial peptides (AMPs) by *C. megacephala* larvae, which exhibit promising applications in wound treatment and infection control [9–11]. This dual utility in waste management and antimicrobial compound production positions *C. megacephala* as a valuable resource in addressing global challenges related to waste and public health.

Despite its potential, research on the bioconversion capabilities of *C. megacephala* larvae remains limited. This study evaluates the efficiency of *C. megacephala* larvae in reducing food waste and explores the antimicrobial properties of their extracts. By addressing these dual benefits, this research contributes to the development of sustainable, environmentally friendly, and economically viable strategies for food waste management, while showcasing the potential biomedical applications of *C. megacephala*-derived products.

## Materials and methods

### Rearing of flies in the laboratory

The *C. megacephala* adults used in this study were obtained from a laboratory-maintained strain. The larvae were reared in a controlled environment at 24–28 ±  0.5°C, 60–70% relative humidity, and a 12-h light/dark cycle, at the Department of Microbiology and Parasitology, Faculty of Medical Science, Naresuan University, Phitsanulok, Thailand. All animal experiments were conducted in strict accordance with the guidelines and regulations approved by the Naresuan University Animal Care and Use Committee (NUACUC, Protocol No. NU-AI670603). For maintaining the fly colony in the laboratory, adults were fed *ad libitum* with fresh pork liver and a 10% (w/v) sugar solution supplemented with 5% (v/v) multivitamin syrup (SEVEN SEAS, Thailand), while larvae were reared with fresh pork liver as previously described [8].

For the bioconversion experiment, an optimized culture medium was prepared with slight modifications to the method described by Niu et al. to support larval development in a cylindrical rearing box with a lid (diameter: 11 cm, height: 12 cm) [6]. The food waste used in this study primarily consisted of carbohydrate- and protein-rich materials (e.g., rice, meat, and vegetable scraps) sourced from a university cafeteria. The waste was initially drained using strainers and then left to settle at room temperature for 2 h. Fresh pork liver or food waste (200 g per group) was used, with 20 g of sawdust added as an adjuvant based on preliminary studies (unpublished data), and 0.15 g of *C. megacephala* eggs was added to each group. Negative controls (without fly eggs) consisted of fresh pork liver or food waste mixed with sawdust in the same ratio (10:1). After the eggs hatched, the culture medium was mixed approximately every 24 h. After five days, the larvae and culture medium were separated, and their weights were measured. Third-instar larvae were then collected for subsequent experiments.

### Preparation of excretory-secretory (ES) products and whole-body extracts (WE) of fly larvae

#### Excretory-secretory (ES) products.

The ES products were prepared using a modified method based on van der Plas et al. [12]. Third-instar larvae were collected from the rearing box, washed with 70% ethanol, and rinsed three times with sterile water. The larvae were distributed into 20 sterile 50 ml centrifuge tubes, each containing 100 larvae in 100 µl of sterile water. The tubes were incubated for 60 min at 24–28°C in the dark. After incubation, the ES products from all 20 tubes were pooled into a sterile centrifuge tube. A protease inhibitor cocktail (AMRESCO, OH, USA) was added to prevent protein/peptide degradation, and the pooled ES products were centrifuged at 1,300 *×  g* for 5 min at 4°C to remove particulate matter. The supernatant was filtered through a 0.45 µm syringe filter for sterilization, aliquoted into sterile microcentrifuge tubes, and stored at -20°C.

#### Whole-body extracts (WE).

The whole-body extracts were prepared using a modified version of the method described by Choi et al. [13]. First, 400 inactivated third instars were homogenized in 200 ml of absolute methanol at a ratio of 2:1. The homogenate was incubated overnight at room temperature with continuous orbital shaking. The mixture was then transferred to centrifuge tubes and centrifuged at 4,000 *×  g* for 30 min at 4°C. The supernatant was collected and transferred to a flask, where it was concentrated using a Hei-VAP Core rotary evaporator (Heidolph, Korea). To further remove residual methanol, the concentrated extracts were subjected to centrifugal evaporator CVE-2200 (Eyela, Japan). The final concentrated extracts were resuspended in 5% DMSO, sterilized through a syringe filter, and 100 µl of the larval extracts were applied to blood agar and brain-heart infusion agar plates. These plates were incubated at 37°C for 48 h to ensure sterility before conducting antimicrobial assays.

### Determination of protein concentration

The protein concentration in the extracts (ES and WE) was determined using the Bradford assay (Bio-Rad, CA, USA), with bovine serum albumin (BSA) as the standard. A 5% DMSO solution was used as the blank for WE, while dH₂O was used as the blank for ES. A series of sample dilutions was prepared, and 160 µl of each dilution was transferred into separate tubes. Subsequently, 40 µl of Coomassie Brilliant Blue reagent was added to each tube. The absorbance of each sample was measured at 595 nm, using SpectraMax^®^ ABS and ABS Plus absorbance microplate readers (Molecular Devices, CA, USA). Protein concentrations were calculated by comparing the absorbance values to a standard curve generated from BSA.

### Antimicrobial susceptibility test

#### Microbial cultures and strains used in the study.

This study employed a variety of microorganisms, including bacteria, yeast, and filamentous fungus, which were provided by the Department of Microbiology and Parasitology, Faculty of Medical Science, Naresuan University, Thailand. The bacterial strains included *Bacillus subtilis* DMST 5871, *Pseudomonas aeruginosa* TISTR No. 1467, *Escherichia coli* TISTR No. 887, *Staphylococcus epidermidis* DMST 15505, *Staphylococcus aureus* TISTR No. 1466, and *Streptococcus pyogenes* DMST 4369. The yeast strains included *Candida albicans* DMST 8684 and *Malassezia furfur* CBS 1878, while the filamentous fungus included *Aspergillus flavus* DMST 22950. Bacterial cultures were grown on nutrient agar, except for *S. pyogenes*, which was cultured on blood agar. All bacterial cultures were incubated at 37°C for 24 h prior to use. Yeast strains were cultured on Sabouraud Dextrose Agar (SDA) at 37°C for 24 h, while filamentous fungus was grown on SDA and incubated at room temperature for 48-72 h. The study was approved by the Naresuan University Institutional Biosafety Committee (NUIBC, Protocol No. MI 66-12-54) to ensure adherence to biosafety standards.

#### Disc diffusion method.

The modified Kirby-Bauer method was employed to evaluate the susceptibility of bacteria and yeast to the larval extracts [14]. Bacterial suspensions were prepared at a McFarland standard 0.5 (10^8^ cells/ml), while yeast suspensions were prepared at a McFarland standard of 1 (10^6^ cells/ml). The suspensions were evenly spread onto Mueller-Hinton agar plates. Subsequently, 100 μg of the tested extracts (ES products or whole-body extracts) was applied to 6.0 mm blank paper disc (Schleicher & Schuell BioScience GmbH) and placed on the agar surface. The plates were incubated at 37°C for 24 h for bacterial cultures and at 30°C for 48 h for yeast cultures. Zones of inhibition were measured using a digital vernier caliper. Antibacterial (tetracycline 30 μg, penicillin 10 μg, ciprofloxacin 5 μg) and antifungal discs (fluconazole 1 μg, zinc pyrithione 20 μg) served as positive controls, while sterile 5% DMSO was used as a negative control. All assays were performed in triplicate, and the procedures were repeated to ensure reliability.

#### Agar toxicity test.

The agar toxicity testing protocol for filamentous fungi (*A. flavus*) was adapted from the methodologies of Armengol et al. and Elizabeth et al. [15,16]. In this test, 20 ml of sterile, warm SDA was mixed with the larval extracts to achieve a final concentration of 100 µg/ml. The mixture was thoroughly vortexed for uniform distribution and then poured into a Petri dish. A colony of *A. flavus*, cut using a 6 mm cork borer, was placed at the center of the SDA plate containing the larval extracts. A growth control of *A. flavus* was placed on a separate SDA plate without the larval extracts, while SDA containing 10 mg of cycloheximide served as the positive control. The plates were incubated at room temperature for 2 to 6 days. After incubation, the growth and morphological characteristics of the *A. flavus* colonies were measured and observed. The percentage of inhibition (% inhibition) was calculated using the formula:


$$\text{\% inhibition}=\text{1}-\left(\frac{\text{Diameter of fungal colony in the test}}{\text{Diameter of fungal colony in the control}}\right)\times \text{100}$$

#### Broth dilution method for MIC and MBC determination.

The resazurin-based turbidometric (TB) assay was used to evaluate the inhibitory effects of ES products and whole-body larval extracts of *C. megacephala* on *P. aeruginosa* and *B. subtilis*. This assay was adapted from the method described by Teh et al. [17]. Broth dilutions were prepared according to the Clinical and Laboratory Standards Institute (CLSI) protocol. The extracts were diluted in Mueller-Hinton Broth (MHB) to obtain final concentrations ranging from 400 to 50 µg/ml. A bacterial suspension, diluted to 10^6^ cells/ml, was added to all tubes except for those designated as broth sterility and larval extracts sterility controls. Each bacterial species was tested in duplicate. Following overnight incubation at 37°C, resazurin solution (6.75 mg/ml) was added to each tube, and the tubes were incubated for an additional 4 h at 37°C. Color changes were monitored and recorded. The Minimum Inhibitory Concentration (MIC) was determined as the lowest concentration at which no color change was observed. To determine the Minimum Bactericidal Concentration (MBC), one microliter of each bacterial dilution was inoculated into nutrient agar plates by using a calibrated loop. After an additional 24 h incubation, the plates were examined for bacterial growth.

### Mass spectrometry

#### In-solution digestion.

Proteins were purified using a Clean-up kit (GE Healthcare, IL, USA) and dissolved in 8 M urea. Protein concentration was determined using the Bradford method (Bio-Rad). For each sample, 30 µg of protein was diluted with 100 mM dithiothreitol in 100 mM TEAB for 30 min at 24–28°C, followed by alkylation with 100 mM iodoacetamide in 100 mM TEAB for an additional 30 min in the dark. The reaction was quenched with the reduction buffer for 15 min, and the proteins were digested with Trypsin Gold (Promega, WI, USA) at 37°C for 16 h. The mixture was dried using a nitrogen evaporator (Organomation, MA, USA), resuspended in 0.1% formic acid, and subsequently purified with a C18 Zip tip. The peptides were dried and stored at -80°C. To measure peptide concentration, the peptides were resuspended in 0.1% formic acid and measured using a NanoDrop 1000 (Thermo Fisher Scientific, Germany).

#### Nano-LC-MS/MS analysis.

Peptides were analyzed using a Nano-LC-MS/MS system, which consisted of a Dionex Ultimate 3000 RSLCnano System (Thermo Scientific) and a CaptiveSpray source/Quadrupole ion trap mass spectrometer (Q-ToF Compact, Bruker, Germany). One µg of peptides was enriched on a Nano trap column and separated on a PepMap100 C18 LC column. Elution was performed with a 2-95% Solvent B gradient over 90 min at a flow rate of 300 nl/min and at 60°C. The mobile phases used were A) 0.1% FA in water and B) 0.08% FA in 80% acetonitrile. The gradient for mobile phase B was as follows: 2% (5 min), 30% (60 min), 50% (10 min), 70% (5 min), 95% (5 min), followed by a rapid decrease to 2% and re-equilibration (5 min). The drying gas was set to 5 l/min at 150°C, with a nebulizer gas pressure of 0.2 bars. MS acquisition was carried out in positive ionization mode at 6 Hz, with a mass range of m/z 150-2200. AutoMSn CID fragmentation was performed at low (4 Hz) and high (16 Hz) rates for the top 2 precursor ions, with a 3-second dynamic exclusion. Peptide sequences were matched against the UniProt-Calliphoridae database using MASCOT (version 2.3). Search parameters included carbamidomethylation of cysteine (fixed modification), oxidation of methionine (variable modification), peptide tolerance of ± 1.6 Da, and MS/MS fragment tolerance of ± 0.8 Da. Proteins with MASCOT scores above the threshold and *p*-values <  0.05 were considered for analysis. This study specifically investigated proteins with molecular weights below 20 kDa, emphasizing their antimicrobial properties.

### Cytotoxicity testing

The cytotoxicity test was performed using a modified method based on Siriwath et al. [18]. Cytotoxic activity was evaluated against HaCaT (immortalized human keratinocyte cells) cells, which were provided by the National Center for Genetic Engineering and Biotechnology (BIOTEC), Thailand. The HaCaT cells were cultured in Dulbecco’s Modified Eagle Medium (DMEM) supplemented with 10% (v/v) fetal bovine serum (FBS) and an antibiotic solution containing 100 units/ml of Antibiotic-Antimycotic (Gibco, Thermo Fisher Scientific, MA, USA). The cells were maintained at 37°C with 5% CO_2_. The effect of ES products and whole-body extracts on cell proliferation was measured using an MTT assay (3-[4,5-dimethylthiazole]-2,5-diphenyltetrazolium bromide). HaCaT cells were seeded in 96-well plates at a density of 2 × 10^3^ cells/well for 24 h before being treated with various concentrations of ES products and whole-body extracts (0, 0.1, 1, 10, and 100 µg/ml). The cells were then incubated for 24 h. A 30% dimethyl sulfoxide (DMSO) solution was used as a positive control. After the incubation periods, 10 µl of MTT was added to each well to achieve a final concentration of 0.5 µg/ml, and the plates were incubated for an additional 3 h at 37°C. Following this, the medium containing MTT was removed, and the formazan crystals were dissolved in DMSO (Sigma-Aldrich, MO, USA). Absorbance was measured at 540 nm, and cytotoxicity was calculated using the formula:


$$\text{\% Cytotoxicity}=\left(\frac{\text{OD of treated cells}}{\text{OD of untreated cells}}\right)\times 100$$

This formula provided a quantitative measure of cell viability in response to the treatments.

### Statistical analysis

The data are expressed as the mean ±  standard deviation (SD) derived from biological triplicate experiments. Statistical analysis was performed using Microsoft Excel for Mac (version 16.6.1, 22101101). The statistical significance of the differences between two different groups was determined with Student’s t-test using GraphPad Prism (version 8). *p* < 0.05 was considered to indicate a statistically significant difference. The significant indicators are *  =  *p* < 0.05, ** =  *p* < 0.01 and *** =  *p* < 0.001.

## Results

### The larvae capacity for reducing food waste

The optimal culture medium ratio was determined to be 10:1 for food waste to adjuvant (sawdust). Under these conditions, 220 g of culture medium (comprising 200 g of food waste or fresh pork liver and 20 g of sawdust) was effectively processed by 0.15 g of *C. megacephala* eggs within five days. The larval survival rate was 85-95% in both groups, with no difference observed between the group fed with food waste and the control group fed with fresh pork liver. The reduction in culture medium and the larval production were compared among four groups. The results indicated that the experimental groups exhibited significantly greater reductions than the negative control groups. The larvae reduced food waste or fresh pork liver approximately three times more effectively than natural decomposition. The bioconversion process yielded an increase of 111.60-fold in fly larvae from food waste and a 153.20-fold increase from fresh pork liver. Additionally, the decomposition of food waste was assessed over a five-day period, both with and without *C. megacephala* larvae. The results demonstrated that the weight of food waste treated with larvae decreased by approximately 21.87%, while the weight of fresh pork liver decreased by 24.82% (Table 1).

**Table 1:** Comparative analysis of bioconversion efficiency of *C. megacephala* on various substrates after five days.

Substrates (ratio)	Fly larvae mass (fold increase)	Reduction of culture medium (%)
**Negative control group 1**	–	7.40
Sawdust: Food waste (1:10)		
**Negative control group 2**	–	7.58
Sawdust: Fresh pork liver (1:10)		
**Positive control group**	153.20	24.82
Eggs: Sawdust: Fresh pork liver (0.075:1:10)		
**Experiment group**	111.60	21.87
Eggs: Sawdust: Food waste (0.075:1:10)		

### Yield and properties of larva extracts

**Fig 1:**
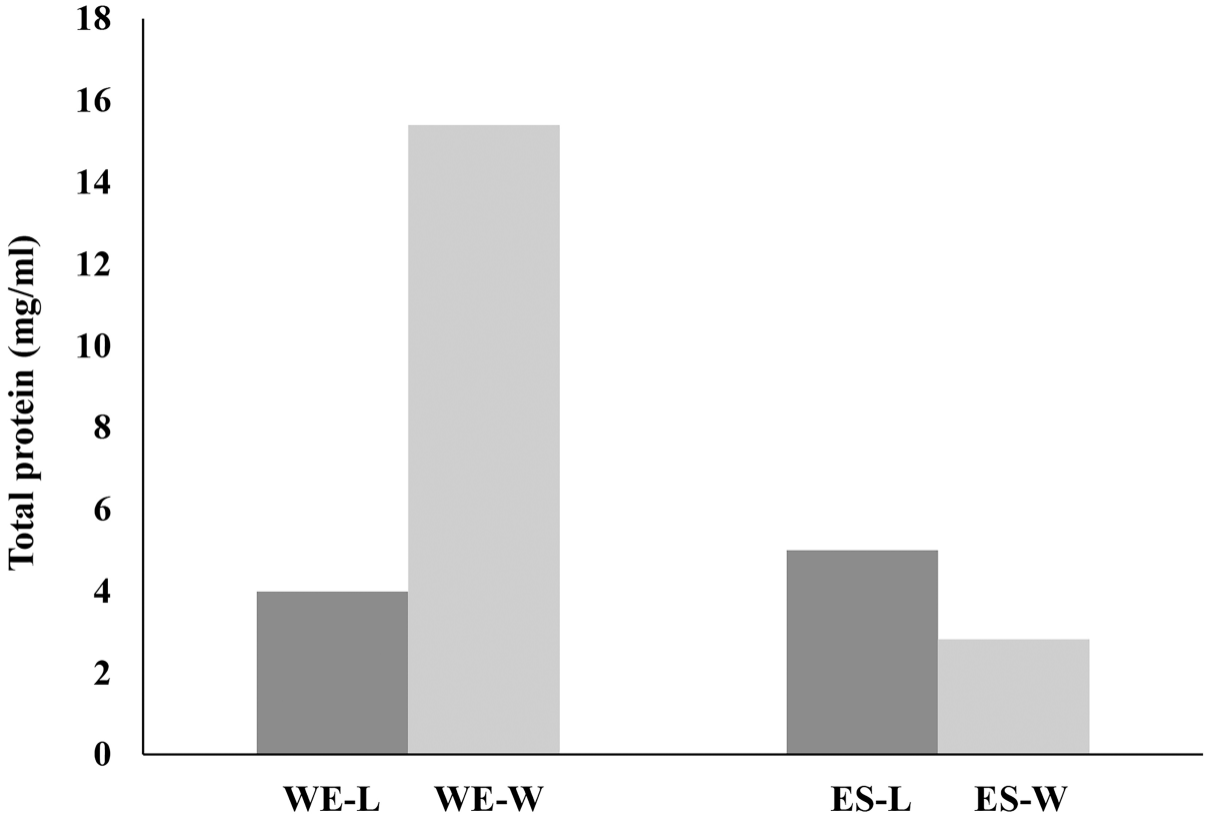
Comparative protein concentrations in extracts from *C. megacephala* larvae rearing with different substrates. This figure displays the protein concentrations measured in milligrams per milliliter (mg/ml) for ES products and whole-body extracts derived from *C. megacephala* larvae. The larvae were reared using two different substrates: fresh pork liver (WE-L, ES-L) and food waste (WE-W, ES-W).

The yield of larval extracts is presented in Table 2. The results indicated that the rearing substrates significantly affected the yield and protein concentration of *C. megacephala* larval extracts. When comparing fresh pork liver and food waste as substrates, it was observed that larvae reared on food waste yielded a greater amount of whole-body extracts (0.80 g or 3.40%) compared to those reared on fresh pork liver (0.60 g or 2.50%). In terms of protein concentration, the total protein content from the whole-body extracts derived from food waste (WE-W) was 15.40 mg/ml, which was markedly higher than that from fresh pork liver (WE-L), at 3.98 mg/ml. Conversely, the ES products exhibited an opposite trend, larvae reared on fresh pork liver produced products (ES-L) with a protein concentration of 4.99 mg/ml, whereas those reared on food waste (ES-W) had a protein concentration of 2.82 mg/ml (Fig 1).

**Table 2:** Yield of whole-body extracts from third-instar *C. megacephala* larvae reared on different substrates.

Parameters	Type of rearing medium	
**Fresh pork liver**	**Food waste**
Methanol solvent volume (ml)	200	200
Number of larvae	400	400
Total weight of larvae (g)	23.50	23.50
Extract weight (g)	0.60	0.80
Yield (% w/w)	2.50	3.40

### Analysis of the antimicrobial activities of larval extracts

The antimicrobial activities of larval extracts from *C. megacephala* were evaluated using the disc diffusion method and the resazurin-based turbidimetric (TB) assay. The disc diffusion method demonstrated that the extracts were effective against *B. subtilis* (Gram-positive bacteria) and *P. aeruginosa* (Gram-negative bacteria) but exhibited no inhibitory effects on yeast and filamentous fungus. This specificity suggests that the extracts may contain components primarily active against bacterial pathogens (Table 3). Further analysis using the broth dilution method, as shown in Table 4, determined that the minimal inhibitory concentration (MIC) for the larval extracts against *B. subtilis* was 100 µg/ml for both extracts derived from larvae reared on food waste and fresh pork liver. Interestingly, the extracts from larvae reared on food waste inhibited *P. aeruginosa* at the same MIC of 100 µg/ml, whereas extracts from larvae fed with fresh pork liver required concentrations exceeding 400 µg/ml, indicating lower efficacy. The ES products from both feeding conditions also inhibited *B. subtilis* at 100 µg/ml but were ineffective against *P. aeruginosa* at 400 µg/ml, as confirmed by the color change from blue to pale pink in the TB assay, indicating bacterial growth. However, the extracts did not demonstrate bactericidal effects, as none of the tested concentrations resulted in complete bacterial elimination (minimum bactericidal concentration >  400 µg/ml). These findings suggest that the extracts exhibit bacteriostatic rather than bactericidal properties.

**Table 3:** Antimicrobial effects of *C. megacephala* extracts and secretory products on bacteria, yeast and filamentous fungus.

Microorganisms	Inhibition zone (mean ± SD; mm)				
**WE-L**	**WE-W**	**ES-L**	**ES-W**	**P**
**Gram-positive bacteria**					
* B. subtilis*	7.15 ± 0.22	7.79 ± 0.20	8.09 ± 0.36	7.96 ± 0.36	32.50 ± 0.71
* S. epidermidis*	0.00 ± 0.00	0.00 ± 0.00	0.00 ± 0.00	0.00 ± 0.00	24.50 ± 0.50
* S. aureus*	0.00 ± 0.00	0.00 ± 0.00	0.00 ± 0.00	0.00 ± 0.00	29.50 ± 0.50
* S. pyogenes*	0.00 ± 0.00	0.00 ± 0.00	0.00 ± 0.00	0.00 ± 0.00	30.00 ± 0.00
**Gram-negative bacteria**					
* P. aeruginosa*	7.15 ± 0.18	7.92 ± 0.41	0.00 ± 0.00	0.00 ± 0.00	14.68 ± 0.28
* E. coli*	0.00 ± 0.00	0.00 ± 0.00	0.00 ± 0.00	0.00 ± 0.00	22.50 ± 0.50
**Yeast**					
* C. albicans*	0.00 ± 0.00	0.00 ± 0.00	0.00 ± 0.00	0.00 ± 0.00	26.00 ± 0.00
* M. furfur*	0.00 ± 0.00	0.00 ± 0.00	0.00 ± 0.00	0.00 ± 0.00	15.50 ± 0.71
**Filamentous fungus**	**% Inhibition**				
	**WE-L**	**WE-W**	**ES-L**	**ES-W**	**P**
*A. flavus*	0	0	0	0	100

**P (Positive control):** For all bacteria tested, tetracycline (30 μg) was used, except for *S. pyogenes* (penicillin, 10 μg) and *P. mirabilis* (ciprofloxacin, 5 μg). For yeast, *C. albicans* was tested with fluconazole (1 μg), and *M. furfur* with zinc pyrithione (20 μg). For filamentous fungus, *A. flavus* was tested with cycloheximide (10 mg).

**Negative control:** 5% DMSO was used.

**WE-L:** whole-body extracts after rearing with fresh pork liver (100 μg).

**WE-W:** whole-body extracts after rearing with food waste (100 μg).

**ES-L:** excretory-secretory products after rearing with fresh pork liver (100 μg).

**ES-W:** excretory-secretory products after rearing with food waste (100 μg).

Data are expressed as mean ±  SD, and each experiment was conducted in triplicate.

**Table 4:** Minimal inhibitory concentration (MIC) of *C. megacephala* whole-body extracts and secretory products using broth dilution.

Microorganisms	Minimal inhibitory concentration (µg/ml)			
**WE-L**	**WE-W**	**ES-L**	**ES-W**
Gram-positive bacteria *B. subtilis*	100	100	100	100
Gram-negative bacteria *P. aeruginosa*	> 400	100	> 400	> 400

The experiments were performed in duplicate and repeated twice

### Protein profile analysis from ES products and whole-body extracts

**Fig 2:**
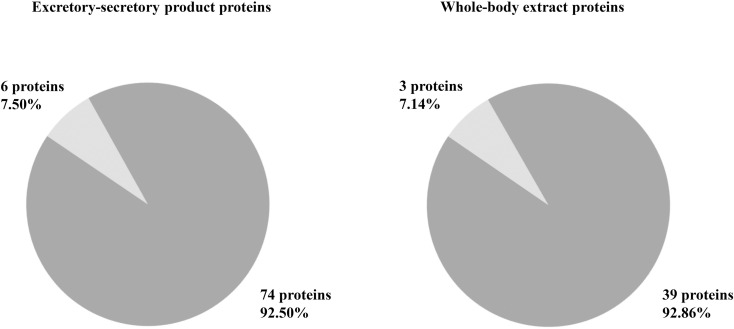
Proteins characterization of *C. megacephala* products. The pie charts represent the analysis results obtained using liquid chromatography-tandem mass spectrometry (LC-MS/MS), combined with data from the NCBI and UniProt-Calliphoridae databases. The charts display the proportions of characterized (dark grey) and uncharacterized (pale grey) proteins in ES products and whole-body extracts from *C. megacephala* larvae.

The proteomic analysis of ES products and whole-body extracts from *C. megacephala* larvae reared on fresh pork liver revealed a detailed protein profile using LC-MS/MS. In the ES products, 80 proteins were identified, of which 74 (92.50%) were characterized and 6 (7.50%) were uncharacterized. Meanwhile, the whole-body extracts contained 42 proteins, with 39 (92.86%) characterized and 3 (7.14%) uncharacterized. These findings indicate a high level of protein characterization in both sample types, with most identified proteins having molecular weights under 20 kDa. Fig 2 illustrates the distribution of characterized and uncharacterized proteins in both sample types. Additionally, S1 Table presents a detailed list of the identified proteins, including their names and sources. These findings indicate the diverse protein composition in both ES products and whole-body extracts, indicating their biological significance and potential applications in further studies.

### Effect of larval extracts on cell viability in HaCaT cells

**Fig 3:**
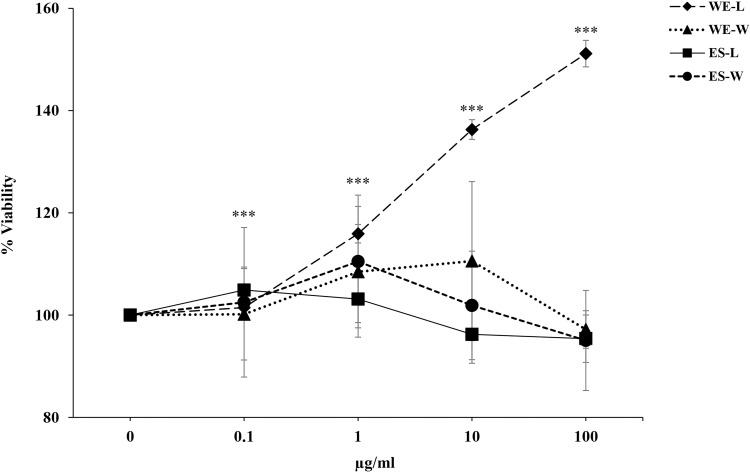
Cell viability response to *C. megacephala* larval extracts. The graph presents the effects of varying concentrations (0.1, 1, 10, 100 µg/ml) of excretory-secretory (ES) products and whole-body extracts (WE) from *C. megacephala* larvae on HaCaT cell viability for 24 h, measured using the MTT assay. The treatments include: WE-L (♦): whole-body extracts from larvae reared on fresh pork liver. WE-W (▲): whole-body extracts from larvae reared on food waste. ES-L (■): excretory-secretory products from larvae reared on fresh pork liver. ES-W (●): excretory-secretory products from larvae reared on food waste. The graph shows the percentage of cell viability, and the error bars represent standard errors within the experimental replicates. The significant indicator is *** =  *p* < 0.001.

The effect of larval extracts on the viability of HaCaT cells was evaluated using a range of concentrations (0.1, 1, 10, and 100 µg/ml) in toxicity assays. An MTT proliferation assay was employed to assess the cytotoxicity of the peptides derived from both ES products and whole-body extracts. As depicted in Fig 3, the extracts maintained cell viability above 90% across all tested concentrations, showing no significant cytotoxicity. The analysis showed that while the amount of formazan produced by living cells varied, there was no significant cytotoxic activity observed, even at the highest concentrations of ES products and whole-body extracts. Remarkably, WE-L extracts significantly increased cell viability at all concentrations (*p* < 0.001) compared to the control, while WE-W, ES-L, and ES-W showed no significant increases.

## Discussion

This study demonstrates the potential of *C. megacephala* larvae for sustainable food waste bioconversion and production of antimicrobial compounds. These bioactive compounds, identified in both ES products and whole-body extracts, enhance the potential applications of larvae in waste management. Despite being widely recognized for its medical significance particularly in its adult stage as a mechanical vector of pathogens, a cause of myiasis, and a valuable species in forensic entomology [8], the bioconversion capabilities of *C. megacephala* have remained largely unexplored. The house fly (*M. domestica*), another medically important species, has been extensively studied for its ability to convert organic waste into high-value biomass [6,19–22]. This study aims to bridge the gap by positioning *C. megacephala* as a promising alternative, offering new insights into its potential for sustainable waste management and antimicrobial production.

The findings of this study confirm the efficiency of *C. megacephala* in reducing food waste, aligning with previous research demonstrating its ability to alter microbiota, reduce pathogenic bacteria, and stabilize microbial communities during composting [23]. These changes, particularly involving genera such as *Pseudomonas* and *Prevotella*, underscore the complex ecological interactions facilitated by *C. megacephala* larvae. Furthermore, the observed reduction in greenhouse gas emissions, including CH₄ and N₂O, highlights the species’ potential in mitigating climate impacts, particularly during the critical early phase of composting [23]. Moreover, *C. megacephala* larvae have been recognized as a potential biodiesel resource [24]. Integrating *C. megacephala* into waste management systems, there is an opportunity to enhance resource efficiency while contributing to broader sustainability goals. However, addressing challenges such as scalability, odor control, larval escape, and biosecurity concerns in diverse settings will be essential for fully realizing this potential. Recent studies have proposed integrating larval bioconversion into circular economy frameworks, where this process not only reduces food waste but also generates valuable byproducts like protein-rich biomass and fertilizers [25–28]. Such approaches could enhance the economic and environmental viability of waste management systems.

In addition to its bioconversion potential, *C. megacephala* exhibits exceptional adaptability to diverse climates and habitats, thriving in both urban and rural areas. Its widespread distribution across tropical and subtropical regions, combined with its ability to utilize various organic substrates, makes it a versatile species for waste management [8]. Despite these advantages, the large-scale application of *C. megacephala* in waste management systems remains underdeveloped. Addressing the challenges of scalability through targeted research and innovation is essential for unlocking its full potential. One of the limitations of bioconversion of food waste by insects is high moisture [6,22]. To address these challenges, low-cost adjuvants such as agricultural waste can be used to improve substrate texture and aeration [7]. In our study, sawdust was used to improve rearing conditions by enhancing substrate porosity and oxygen availability, which facilitated larval activity and accelerated waste degradation. This approach demonstrates the potential of bioconversion systems for sustainable food waste management, effectively processing organic waste while optimizing conditions for larval development. Using food waste as a substrate not only supports waste reduction but also improves biomass conversion efficiency, making it a valuable option for resource utilization. The integration of sawdust further enhances the system’s performance, showing how simple adjustments can significantly advance waste management and sustainability.

Larval extracts show strong potential in medical applications, particularly in promoting cell growth and aiding wound healing. Their antimicrobial activity against *B. subtilis* and *P. aeruginosa* underscores their applicability in developing novel treatments for bacterial infections. The lack of bactericidal effects in the larval extracts may be attributed to their mode of action, which primarily inhibits bacterial growth rather than directly killing bacteria. Despite this limitation, the bacteriostatic activity could still hold significant potential in applications where growth suppression is sufficient, such as preventing bacterial colonization in wounds or serving as a supplementary treatment alongside other antimicrobial [29]. To further expand their utility, future studies could explore modifications or combinations of these extracts to enhance their bactericidal properties. Consistent with expectations, the larval extracts revealed no inhibitory effects on fungi, including *A. flavus*, *C. albicans*, and *M. furfur*, highlighting the specificity of their active compounds for bacterial pathogens. This observation aligns with previous findings by Suriyakan et al., which reported no antifungal activity of excretory and secretory products from *C. megacephala* larvae [11]. In addition to their antimicrobial activity, larval extracts also offer promising opportunities in agricultural and livestock industries. Lee et al. demonstrated that defatted *H. illucens* larvae extracts serve as natural antibiotics and feed additives for livestock, reducing reliance on conventional antibiotics and addressing the pressing issue of antibiotic resistance [30]. Moreover, feeding the larvae organic waste not only enhances their sustainability as an agricultural resource but also highlights their economic viability. For instance, crude-oil-extracted *H. illucens* powder could act as a cost-effective alternative to synthetic antimicrobials like melittin, making it a practical solution for large-scale use.

Larval extracts from *C. megacephala* show strong potential for maggot debridement therapy (MDT) due to their wound-healing, antimicrobial properties, and non-toxic effects on HaCaT cells, human keratinocytes commonly used to study skin biology. These findings align with previous research, such as the study by Suriyakan et al., which demonstrated the antimicrobial properties of *C. megacephala* excretions and secretions against *P. aeruginosa*, a key pathogen in chronic wounds and antibiotic resistance [11,31]. Similarly, the sarconesin II fraction from *Sarconesiopsis magellanica* (Diptera: Calliphoridae) exhibited a lack of cytotoxicity [32], further supporting the potentail use of blow fly-derived extracts in wound healing therapies [33]. In addition to their non-toxic profile, *C. megacephala* larval extracts promote cell growth *in vitro*, a regenerative property critical for MDT, where rapid tissue repair is essential. This observation is consistent with studies by Lu et al., which identified an ATPase inhibitory peptide from *C. megacephala* larvae that effectively inhibits *P. aeruginosa* growth. These findings establish the therapeutic potential of specific larval peptides in targeting resistant bacterial strains and advancing wound healing therapies, addressing the urgent need for new antimicrobial agents [34,35].

Larval extracts from *C. megacephala* are rich in proteins, including those under 20 kDa, involved in detoxification and antimicrobial defense (S1 Table). Among these is a potential multidrug resistance-associated protein that may enhance the larvae’s antimicrobial properties [9]. The protein composition varies between ES products and whole-body extracts, reflecting differences in both their composition and function. ES products contain a broader array of proteins specialized for interacting with the larvae’s external environment, such as thioredoxin domain-containing proteins. Thioredoxins in *C. megacephala* exhibit diverse roles in detoxification, oxidative stress protection, antimicrobial defense, and reproductive development with their structural diversity and functional specificity underscoring their potential as targets for antimicrobial agents or therapeutic interventions [36]. Additionally, heat shock protein 70 (Hsp70) in *C. megacephala* is known for its roles in protein stabilization, folding, oxidative stress protection, and modulation of immune pathways, supporting cellular resilience under environmental stress [37]. In contrast, whole-body extracts are rich in structural proteins that play essential roles in maintaining cellular integrity and supporting various biological functions. Among these proteins, a putative aminoacyl tRNA synthase complex-interacting multifunctional protein was also identified. In *Drosophila melanogaster*, these proteins, particularly members of the phosducin-like protein (PhLP) family, exhibit multifunctional roles, share structural similarities with thioredoxins, and perform chaperone-assisted functions in protein stability and cellular integrity, contributing to cellular resilience under stress conditions [38].

**Fig 4:**
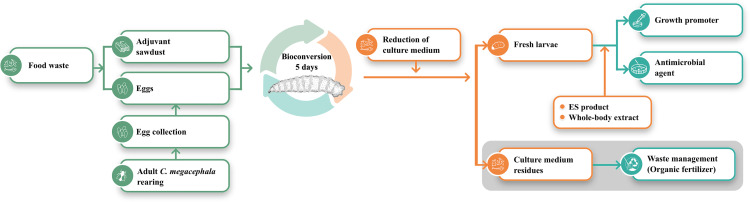
Schematic diagram of the bioconversion process using *C. megacephala.* This diagram illustrates the efficiency of *C. megacephala* in transforming food waste into valuable products. Within a five-day period, food waste mixed with sawdust supports larval development, producing ES products and whole-body extracts. These byproducts have potential applications as growth promoters and antimicrobial agents, while the remaining residue can be further processed into organic fertilizer. Potential applications, marked in the grey box, represent areas for future research not explored in this study. This bioconversion process underscores the potential of *C. megacephala* as a sustainable solution for waste management and biotechnological applications.

The bioconversion process by *C. megacephala* larvae, as shown in the diagram (Fig 4), represents an innovative approach to managing food waste by converting it into valuable byproducts within only five days. This system not only effectively reduces the volume of food waste but also recycles it into useful products such as organic fertilizer and protein-rich larvae, which can be used in animal feed or further processed for pharmaceutical applications, where larval extracts serve as antimicrobial agents and growth promoters. The incorporation of sawdust as an adjuvant is hypothesized to optimize conditions for larval growth and enhance waste reduction efficiency, suggesting its potential to contribute to sustainable waste management and support circular economy initiatives. This approach not only addresses environmental issues related to waste disposal but also creates economic value by converting waste into value-added products, demonstrating a practical application of waste-to-value strategies.

## Conclusions

This study demonstrates that bioconversion of food waste by *C. megacephala* larvae is an effective method for managing protein-based waste. The use of larvae significantly reduces the weight and volume of food waste, providing an environmentally friendly alternative to traditional disposal methods. The inclusion of adjuvants such as sawdust is crucial for optimizing rearing conditions by reducing moisture content and improving the aeration of the waste substrate. Larval extracts from *C. megacephala* exhibit antimicrobial properties and promote cell growth, suggesting potential applications in the pharmaceutical, agricultural, and animal feed industries. These bioactive byproducts offer opportunities for the development of new antimicrobial agents and nutritional supplements for livestock. Despite these advantages, several challenges must be addressed for large-scale application. Issues such as odor control, preventing larval escape, and maintaining process efficiency require further research. Enhancing the efficiency and safety of the bioconversion process is essential for its broader adoption as a sustainable waste management strategy. The large-scale implementation of *C. megacephala* larvae in food waste management could play a crucial role in circular economy models, reducing reliance on landfills and enhancing resource sustainability.

## Supporting information

S1 Table Protein analysis. Characterized proteins identified in excretory-secretory products and whole-body extracts from *Chrysomya megacephala* larvae (UniProt-Calliphoridae database, MASCOT analysis).
(PDF)
