# Supplementary material for: Bioconversion of food waste by Chrysomya megacephala (Diptera: Calliphoridae) larvae: Potential for sustainable waste management and antimicrobial applications
Source: PLoS One. 2025 Apr 15;20(4):e0320747. doi: 10.1371/journal.pone.0320747 (PMC11999131; doi:10.1371/journal.pone.0320747)
Supplement: S1 Table — Characterized proteins identified in excretory-secretory products and whole-body extracts from Chrysomya megacephala larvae (UniProt-Calliphoridae database, MASCOT analysis). (PDF) [file pone.0320747.s001.pdf]

S1 Table. Protein analysis. Characterized proteins identified in excretory-secretory products and whole-body extracts from *C. megacephala* larvae (UniProt-Calliphoridae database, MASCOT analysis).

No.	Excretory-secretory products	Whole body extracts
1	Actin-related protein 3 (A0A0L0BV06_LUCCU)	40S ribosomal protein S10b (A0A0L0BT67_LUCCU)
2	Aminopeptidase (Q8MUI7_LUCCU)	AAA+ ATPase domain-containing protein (A0A0L0BKI7_LUCCU)
3	Apolipophorin (Fragment) (A0A0L0BWP2_LUCCU)	Actin-57B (A0A0L0CFH3_LUCCU)
4	Arylphorin (O18321_CALVI)	A-kinase anchor protein 2 C-terminal domain-containing protein (A0A0L0C6X1_LUCCU)
5	ATPase ASNA1 homolog (A0A0L0C875_LUCCU)	Arginine/serine-rich protein PNISR (A0A0L0BNV1_LUCCU)
6	Axonemal 84 kDa protein (A0A0L0C8R9_LUCCU)	ATP-dependent DNA helicase 2 subunit 1 (A0A0L0BR29_LUCCU)
7	Bifunctional purine biosynthesis protein ATIC (A0A0L0CQM5_LUCCU)	Cadherin-related tumor suppressor (A0A0L0C4R4_LUCCU)
8	Brahma-associated protein of 60 kDa (A0A0L0CCT4_LUCCU)	Charged multivesicular body protein 4b (A0A0L0CFW8_LUCCU)
9	Brain-specific angiogenesis inhibitor 1-associated protein 2 (A0A0L0C0B0_LUCCU)	COP9 signalosome complex subunit 3 (A0A0L0CL66_LUCCU)
10	C2H2-type domain-containing protein (A0A0L0CQR8_LUCCU)	DUF4766 domain-containing protein (A0A0L0CA86_LUCCU)
11	Cadherin-related tumor suppressor (A0A0L0C4S2_LUCCU)	E3 ubiquitin-protein ligase HRD1 (A0A0L0BSZ5_LUCCU)
12	Calponin-homology (CH) domain-containing protein (A0A0L0CDW1_LUCCU)	Elongation factor 1-alpha (Fragment) (L7NUV6_9MUSC)
13	Carbamoyl phosphate synthetase (Fragment) (A0A3T0V880_9MUSC)	Elongation factor 2 (A0A0L0C5C9_LUCCU)
14	Carboxylesterase type B domain-containing protein (A0A0L0BPU1_LUCCU)	Elongation factor Tu (A0A0L0C7B8_LUCCU)
15	CD109 antigen (A0A0L0BSC0_LUCCU)	General transcription and DNA repair factor IIH helicase subunit XPB (A0A0L0BR20_LUCCU)
16	Chaoptin (A0A0L0CB48_LUCCU)	Geranylgeranyl transferase type-1 subunit beta (A0A0L0C702_LUCCU)
17	Chitinase-like protein (A0A2P0P9I0_CHRMG)	Glutamine synthetase (A0A0L0CBW9_LUCCU)
18	Chitin-binding type-2 domain-containing protein (A0A0L0BTH8_LUCCU)	Guanylate kinase/L-type calcium channel beta subunit domain-containing protein (A0A0L0BZB7_LUCCU)
19	CN hydrolase domain-containing protein (A0A0L0CLU6_LUCCU)	Ionotropic glutamate receptor C-terminal domain-containing protein (A0A0L0CH38_LUCCU)
20	c-SKI SMAD4-binding domain-containing protein (A0A0L0CQJ5_LUCCU)	Kazal-like domain-containing protein (A0A0L0BU01_LUCCU)
21	Cupin-like domain-containing protein (Fragment) (A0A0L0BU86_LUCCU)	Long-chain fatty acid transport protein 4 (A0A0L0BRG2_LUCCU)
22	Cyclic nucleotide-binding domain-containing protein (A0A0L0C0W3_LUCCU)	Moesin/ezrin/radixin homolog 1 (A0A0L0BYZ6_LUCCU)
23	DNA polymerase epsilon catalytic subunit (A0A0L0CM66_LUCCU)	Myosin-2 essential light chain (A0A0L0CB99_LUCCU)
24	DNA topoisomerase (Fragment) (A0A0L0CIM9_LUCCU)	NudC domain-containing protein 1 (A0A0L0CCE6_LUCCU)
25	DNA topoisomerase I (A0A0L0C2C6_LUCCU)	Phosphoenolpyruvate carboxykinase (GTP) (A0A0L0BSP4_LUCCU)
26	DUF4201 domain-containing protein (A0A0L0BYP8_LUCCU)	Polyadenylate-binding protein 2 (A0A0L0BPH2_LUCCU)
27	DUF4776 domain-containing protein (A0A0L0C7G4_LUCCU)	Protein kinase domain-containing protein (Fragment) (A0A0L0CEN7_LUCCU)
28	DUF753 domain-containing protein (A0A0L0CHW7_LUCCU)	Putative aminoacyl tRNA synthase complex-interacting multifunctional protein 2 (A0A0L0C8J0_LUCCU)
29	Eukaryotic translation initiation factor 5B (A0A0L0CCN1_LUCCU)	Putative ribosomal RNA processing protein 1 (A0A0L0C1Q2_LUCCU)
30	Fasciclin-1 (A0A0L0CGP3_LUCCU)	Reticulon-like protein (A0A0L0BS32_LUCCU)
31	Glutamyl aminopeptidase (Fragment) (A0A0L0CE23_LUCCU)	RNA-binding protein squid (A0A0L0C0Q7_LUCCU)
32	Glycerophosphocholine phosphodiesterase GPCPD1 (A0A0L0C8G0_LUCCU)	Selenoprotein F/M domain-containing protein (A0A0L0BQ78_LUCCU)
33	Glycogenin-1 (A0A0L0C390_LUCCU)	Serine/threonine-protein kinase Doa (A0A0L0BYF8_LUCCU)
34	GTPase-activating protein (A0A0L0CI42_LUCCU)	Tropomyosin-1, isoforms 9A/A/B (A0A0L0CPH6_LUCCU)
35	Guanylate cyclase (A0A0L0C8M8_LUCCU)	Twitchin (A0A0L0C2P9_LUCCU)
36	Heat shock protein 70 (R4NV57_CHRMG)	UBP-type domain-containing protein (A0A0L0CDD2_LUCCU)
37	histone acetyltransferase (A0A0L0BL87_LUCCU)	Unc-80-like protein (A0A0L0C2W3_LUCCU)
38	Ion transport domain-containing protein (A0A0L0CG13_LUCCU)	UPF0506 domain-containing protein (A0A0L0CGG8_LUCCU)
39	Kazal-like domain-containing protein (Fragment) (A0A0L0CCU4_LUCCU)	Virion RNA polymerase (A0A0L0CE10_LUCCU)
40	Laminin subunit alpha (A0A0L0CCW4_LUCCU)	
41	La-related protein (Fragment) (A0A0L0CFI2_LUCCU)	
42	Larval serum protein 1 beta chain (A0A0L0CA19_LUCCU)	
43	Larval serum protein 2 (A0A0L0CE13_LUCCU)	
44	MADF domain-containing protein (A0A0L0CA34_LUCCU)	
45	MOXD1-like protein 1 (A0A0L0CIZ4_LUCCU)	
46	Odorant-binding protein (L8B8J6_9MUSC)	
47	Outer dense fiber protein 3 (A0A0L0CQB8_LUCCU)	
48	PDZ domain-containing protein 8 (A0A0L0BU58_LUCCU)	
49	Peptidase M14 carboxypeptidase A domain-containing protein (A0A0L0CND6_LUCCU)	
50	Peptidase S1 domain-containing protein (A0A0L0CER1_LUCCU)	
51	Pheromone-binding protein-related protein 2 (A0A0L0C3V5_LUCCU)	
52	Phosphatidylinositol-3-phosphate phosphatase (A0A0L0CN55_LUCCU)	
53	Phosphoribosylformylglycinamidine cyclo-ligase (Fragment) (K7YYY2_COCMA)	
54	Pickpocket protein 28 (A0A0L0CBH7_LUCCU)	
55	Protein jagunal (A0A0L0CCN4_LUCCU)	
56	Protein kinase domain-containing protein (Fragment) (A0A0L0BUU2_LUCCU)	
57	Protein kinase domain-containing protein (A0A0L0CHH2_LUCCU)	
58	Protein takeout (A0A0L0BMU2_LUCCU)	
59	Putative 28S ribosomal protein S25, mitochondrial (A0A0L0C4V6_LUCCU)	
60	Rho guanine nucleotide exchange factor 7 (Fragment) (A0A0L0BSK0_LUCCU)	
61	RNA helicase (A0A0L0CKH7_LUCCU)	
62	Signal peptide peptidase-like 3 (A0A0L0CPA6_LUCCU)	
63	Sodium channel protein (A0A0L0BP99_LUCCU)	
64	Sorbitol dehydrogenase (A0A0L0BYI5_LUCCU)	
65	Succinate dehydrogenase [ubiquinone] flavoprotein subunit, mitochondrial (A0A0L0BSS9_LUCCU)	
66	SUI1 domain-containing protein (A0A0L0BTB1_LUCCU)	
67	Thioredoxin domain-containing protein (A0A0L0BZQ7_LUCCU)	
68	THO complex subunit 5 (A0A0L0CIP9_LUCCU)	
69	Tiggrin (A0A0L0BRQ0_LUCCU)	
70	Transferrin (A0A0L0C0K4_LUCCU)	
71	UBC core domain-containing protein (A0A0L0CD73_LUCCU)	
72	U-box domain-containing protein (A0A0L0CN62_LUCCU)	
73	WD repeat-containing protein 55 homolog (A0A0L0CS07_LUCCU)	
74	ZAD domain-containing protein (A0A0L0CBX7_LUCCU)	

Abbreviations for organisms used in protein analysis:

LUCCU: *Lucilia cuprina*

CHRMG: *Chrysomya megacephala*

CALVI: *Calliphora vicina*

COCMA: *Cochliomyia macellaria*

9MUSC: suborder Muscomorpha
